# Discrimination of ground‐glass nodular lung adenocarcinoma pathological subtypes via transfer learning: A multicenter study

**DOI:** 10.1002/cam4.6402

**Published:** 2023-09-18

**Authors:** Chun‐Long Fu, Ze‐Bin Yang, Ping Li, Kang‐Fei Shan, Mei‐Kang Wu, Jie‐Ping Xu, Chi‐Jun Ma, Fang‐Hong Luo, Long Zhou, Ji‐Hong Sun, Fen‐Hua Zhao

**Affiliations:** ^1^ Department of Radiology Affiliated Dongyang Hospital of Wenzhou Medical University Dongyang China; ^2^ Department of Radiology, Sir Run Run Shaw Hospital Zhejiang University School of Medicine Hangzhou China; ^3^ Department of Radiology Jiaxing Hospital of Traditional Chinese Medicine Jiaxing China; ^4^ Key Laboratory of Diagnosis and Treatment of Digestive System Tumors of Zhejiang Province Ningbo China; ^5^ Cancer Center Zhejiang University Hangzhou China

**Keywords:** adenocarcinoma, deep learning, lung, prognosis, X‐ray computed tomography

## Abstract

**Background:**

The surgical approach and prognosis for invasive adenocarcinoma (IAC) and minimally invasive adenocarcinoma (MIA) of the lung differ. However, they both manifest as identical ground‐glass nodules (GGNs) in computed tomography images, and no effective method exists to discriminate them.

**Methods:**

We developed and validated a three‐dimensional (3D) deep transfer learning model to discriminate IAC from MIA based on CT images of GGNs. This model uses a 3D medical image pre‐training model (MedicalNet) and a fusion model to build a classification network. Transfer learning was utilized for end‐to‐end predictive modeling of the cohort data of the first center, and the cohort data of the other two centers were used as independent external validation data. This study included 999 lung GGN images of 921 patients pathologically diagnosed with IAC or MIA at three cohort centers.

**Results:**

The predictive performance of the model was assessed using the area under the receiver operating characteristic curve (AUC). The model had high diagnostic efficacy for the training and validation groups (accuracy: 89%, sensitivity: 95%, specificity: 84%, and AUC: 95% in the training group; accuracy: 88%, sensitivity: 84%, specificity: 93%, and AUC: 92% in the internal validation group; accuracy: 83%, sensitivity: 83%, specificity: 83%, and AUC: 89% in one external validation group; accuracy: 78%, sensitivity: 80%, specificity: 77%, and AUC: 82% in the other external validation group).

**Conclusions:**

Our 3D deep transfer learning model provides a noninvasive, low‐cost, rapid, and reproducible method for preoperative prediction of IAC and MIA in lung cancer patients with GGNs. It can help clinicians to choose the optimal surgical strategy and improve the prognosis of patients.

## Graphical abstract

This paper reports the development and validation of a three‐dimensional (3D) deep transfer learning model for the discrimination of two ground‐glass nodule (GGN) cancer subtypes, invasive adenocarcinoma (IAC), and minimally invasive adenocarcinoma (MIA), from computed tomography (CT) images.

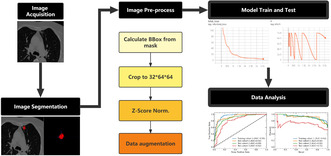

## 1 INTRODUCTION

Lung cancer is a common and highly prevalent malignancy. It is the leading cause of cancer‐related deaths worldwide and has the most rapidly increasing prevalence and mortality among human malignancies.^1^, ^2^ Lung adenocarcinoma is the most common pathological type of lung cancer, accounting for almost half of the total prevalence of all lung cancers. The 2015 World Health Organization histological classification of lung tumors divides lung adenocarcinoma into three main categories: preinvasive lesions, minimally invasive adenocarcinoma (MIA), and invasive adenocarcinoma (IAC). The preinvasive lesions include atypical adenomatous hyperplasia (AAH) and adenocarcinoma in situ (AIS).^3^, ^4^

Clinical management strategies, surgical approaches, postoperative treatment options, and survival rates vary among different subtypes of adenocarcinoma.^5^, ^6^ Preinvasive lesions and MIA tend to have a mild biological behavior and grow slowly, with no significant changes during long‐term follow‐up. They can either be followed up in view of the patient's condition or surgically treated at the optimal time, with a 10‐year disease‐specific survival (DSS) of 100% or 97.3%. Conversely, IAC requires timely surgical treatment, with a lower 10‐year DSS of 74.8% or 80.2%.^7^ This difference in prognosis necessitates the selection of different clinical diagnostic and treatment options as appropriate.

In general, preinvasive lesions and MIA are suitable for planned follow‐up or sublobar resection (wedge resection or segmentectomy) to maximize the preservation of healthy lung tissue and thus reduce surgery‐related mortality and morbidity. By contrast, IAC patients must undergo lobectomy and mediastinal lymph node dissection, with reports stating that postoperative adjuvant therapy can improve their survival.^6^, ^8^, ^9^ Considering these differences in follow‐up strategy, surgical approach, postoperative treatment, and prognosis, preoperative identification of IAC versus MIA preinvasive lesions is essential. However, IAC and MIA manifest as similar pure ground‐glass nodules (GGNs) or mixed GGNs in computed tomography (CT) images, and it is thus difficult to discriminate them accurately based on traditional image features such as size, density, morphology, and blood supply characteristics.

Radiomics is an emerging technology that first transforms medical images into extractable high‐dimensional data and then performs feature extraction, dimensionality reduction analysis, and model development. This allows it to effectively overcome the obstacle to quantitative assessment of tumor heterogeneity, and thus it has significant clinical importance.^10^ However, the feature extraction method in radiomics is manually designed and may not be suitable for better quantitative assessment owing to the limitations of current knowledge and experience.

Recently, a novel deep learning (DL) technique has exhibited outstanding performance in many medical image analysis fields.^11^ Specifically, it first transforms medical image data into high‐resolution feature space data by automatically extracting relevant quantitative and mineable features through DL models and then quantifies tumor heterogeneity through neural networks, thereby providing more information than conventional methods. This DL technique is independent of the level of expertise and is not subject to the inherent limitations of subjective analysis and traditional image interpretation. Studies have demonstrated the efficacy of DL models in solving various clinical problems, such as predicting the aggressiveness of pulmonary GGNs, screening patients for molecular targeted therapy and immunotherapy, classifying metastatic recurrences, and predicting survival rates.^6^, ^12^, ^13^

Although a few studies have applied DL to the prediction of pathological subtypes of GGN‐type lung adenocarcinoma, it has predominantly been used to discriminate between invasive adenocarcinoma (MIA/IAC) and preinvasive lesions (AAH/AIS). This study was conducted to develop a 3D DL‐based model for discriminating between the MIA and IAC GGN subtypes. Specifically, we combined a 3D medical image pretrained model with a fusion model via transfer learning and validated the predictive and discriminative performance of the combined DL‐based model for IAC and MIA.

## 2 MATERIALS AND METHODS

### 2.1 Study population

In this retrospective study, the CT GGN images of patients who were pathologically diagnosed with IAC or MIA were collected from the picture archiving and communication systems of three cohort centers. The inclusion criteria for patients were as follows: (1) pathological diagnosis of pulmonary IAC or MIA by surgical resection or percutaneous needle biopsy; (2) subjected to thin‐slice CT scans with slice thickness of ≤1 mm; (3) underwent chest high‐resolution CT (HRCT) scans within 1 month before surgery and had GGNs in CT images; and (4) did not receive therapy before HRCT scans. Those with significant respiratory motion artifacts or incomplete clinical information were excluded. The local institutional review board approved this retrospective study and waived the requirement for written informed consent.

**FIGURE 1:**
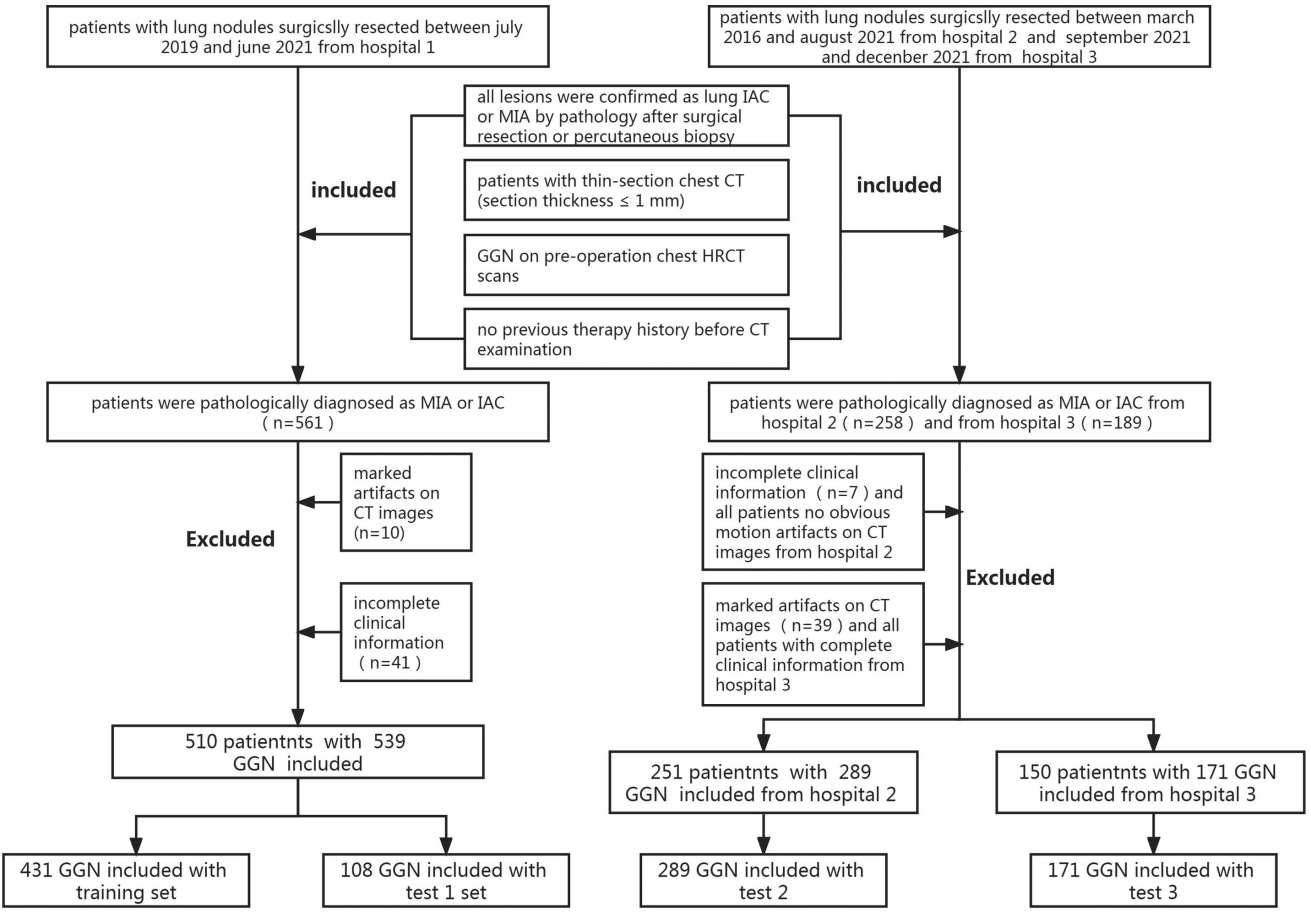
Illustration of inclusion of patients in the study.

Multiple nodules existed in some patients, with each nodule possibly of a different pathological type. Therefore, each GGN was analyzed separately in this scenario. A total of 911 patients (age: 25 to 86 years; mean age: 58.1 ± 18.4 years) met the inclusion criteria and were included, with a total of 999 pulmonary GGNs, of which 387 lesions were located in the left lung (263 in the upper left lobe and 124 in the lower left lobe), and 612 in the right lung (362 in the upper right lobe, 98 in the middle right lobe, and 152 in the lower right lobe). All patients underwent video‐assisted thoracoscopic surgery, 384 patients underwent lobectomy, 615 patients underwent sublobar resection (233 in the segmental resection and 382 in the wedge resection).The 539 GGNs of the first center (Affiliated Dongyang Hospital of Wenzhou Medical University, July 2019–June 2021) were randomly divided into training and internal validation groups in an 8:2 ratio. The 289 GGNs of the second center (Yiwu Tianxiang Medical Eastern Hospital, March 2016–August 2021) and 171 GGNs of the third center (Sir Run Run Shaw Hospital, Zhejiang University School of Medicine, September–December 2021) were used as two independent external validation groups. The workflow is depicted in Figure 1.

### 2.2 Inspection methods

At each center, all CT scans were breath‐hold scans performed at the end of deep inspiration while the patients were in a supine position with the head advanced. The scan range covered the entire area from the lung apex to the lung base. The following CT scanners were used: Aquilion ONE (Canon), Brilliance (Philips), Force (Siemens), Definition (Siemens), and uCT 510 (United Imaging). The scan parameters for each CT scanner are presented in Table 1.

**TABLE 1:** Image acquisition parameters.

Machine type	Canon Aquilion ONE	Philips Brilliance	Siemens Force	Siemens Definition	United Imaging uCT 510
Row	320	64	96	64	16
Tube voltage(KV)	120	120	120	120	120
Effective power of tube (MA)	150	150	Auto	Auto	Auto
Matrix	512 × 512	1024 × 1024	512 × 512	512 × 512	512 × 512
Slice thickness(mm)	0.67	0.67	1.0	1.0	1.0

### 2.3 Image delineation and data preprocessing

All cross‐sectional digital imaging and communications in medicine (DICOM) images of pulmonary GGNs were imported into the software ITK‐SNAP (version 3.8; www.itksnap.org).^14^ This was followed by manual delineation of the regions of interest (ROIs) on pixels layer by layer along the inner edge of the pulmonary GGNs. Subsequently, the ROIs were fused and saved as a 3D image. ROI delineation was performed by four physicians with more than 5 years of experience and then reviewed by a physician with more than 10 years of experience. Large vessels and bronchi were excluded from the ROIs. Consistency analysis was performed using the intraclass correlation coefficient (ICC), with ICC >0.8 indicating good consistency.

**FIGURE 2:**
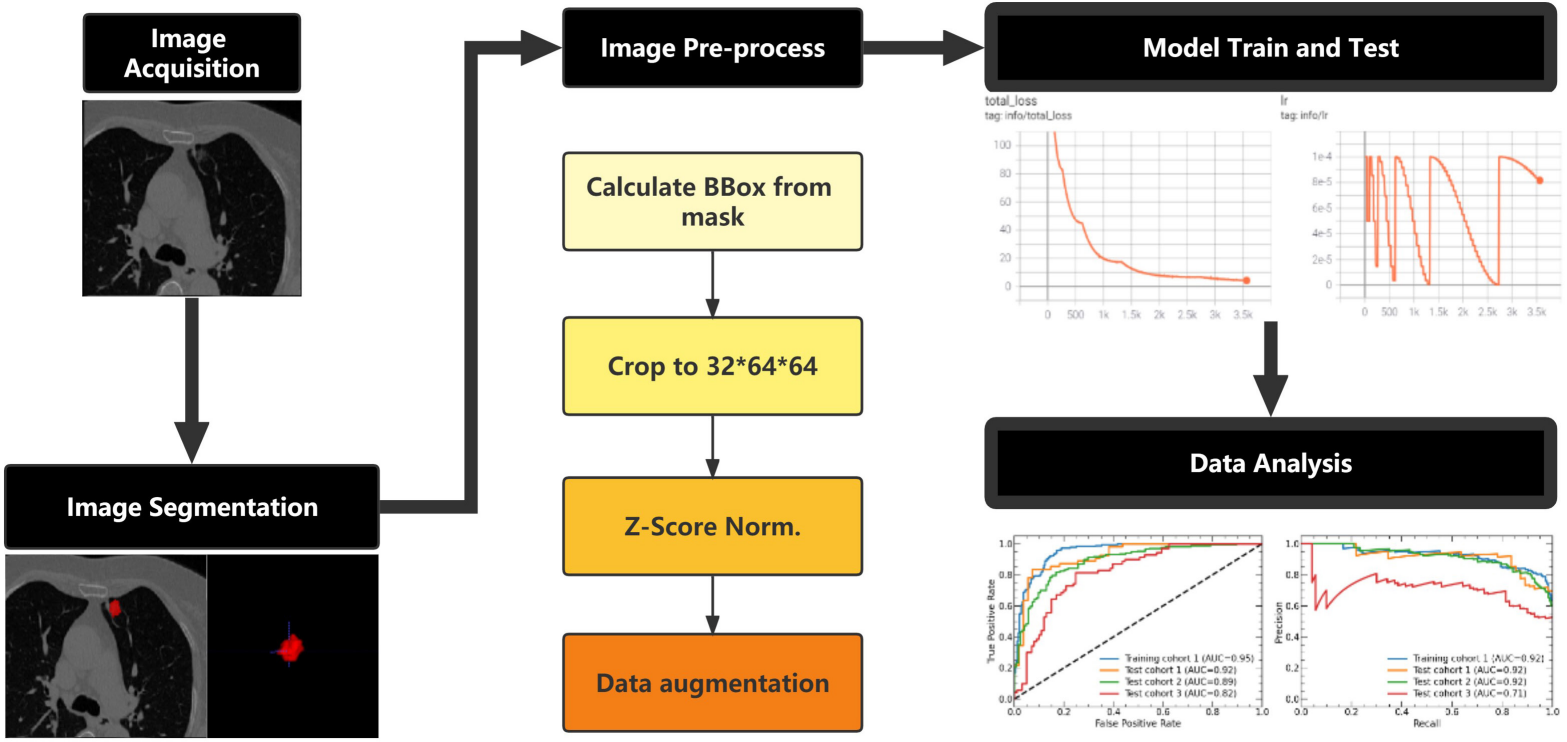
Overall research design.

After image delineation, We cropped the CT images using the lung window, which in our case is [−1200, 500]. Then, the DICOM image of each patient was resampled to a spatial resolution of 0.585 × 0.585 × 1.0 mm, followed by Z‐score normalization, exclusion of data outside four times the standard deviation, and then applied min‐max normalization (normalize to 0–1) of the image values to meet the input requirements of the model. After the preprocessing step, the CT images and delineated image were stitched on the channel, and an image block of size 32 × 64 × 64 centered on the nodule was truncated to be used as the input to the model. Data augmentation was performed during model training by randomly flipping the image block in the horizontal and vertical directions. The overall research design is illustrated in Figure 2.

### 2.4 DL model

**FIGURE 3:**
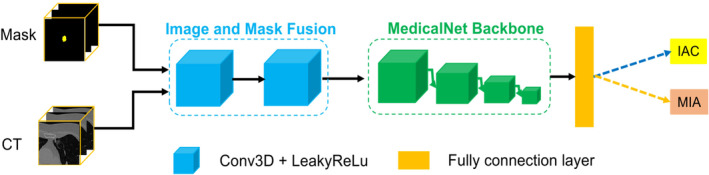
Framework of the 3D deep learning (DL) model.

In this study, a DL model was constructed using a 3D medical image pretrained model (MedicalNet)^15^ and transfer learning. The overall framework is schematically illustrated in Figure 3. MedicalNet was proposed by Tencent in 2019 and was built using numerous medical images. It is the first pretrained 3D DL model specifically for medical images. Because it is built from a large volume of medical image data, it has strong feature extraction capability and thus can be effectively transferred to various medical image‐related tasks.

In this study, the ResNet10 network of MedicalNet was used as the backbone of the feature encoder and the output channel of the last fully connected layer of the original network was set to two. To meet the requirement that the input to MedicalNet be a single‐channel input, after the CT images and delineated image were input to the model, the two input channels were converted to a single channel by passing them through two image‐mask module layers, with each layer consisting of a 3D convolutional layer and a Leaky ReLU activation function.

The network was initialized using the pre‐training weights of MedicalNet and optimized using the Adam optimizer and focal loss function, with the ratio of the learning rate of the MedicalNet backbone to the learning rate of all other layers set to 0.01:1. An stochastic gradient descent with warm restarts strategy was adopted to adjust the learning rate; specifically, the initial value was set to 0.02, and after n epochs, a hot restart and resetting of the learning rate to its initial value were carried out. This cleared the momentum buffer of the optimizer and created a temporary period of instability so that it could escape from suboptimal local minima.^16^ The batch size used for network training was 16, and the model input size was 32 × 64 × 64. The entire network procedure was implemented using the PyTorch DL framework, and all experiments were conducted on an NVIDA GeForce RTX 3090 GPU.

### 2.5 Statistical analysis

Descriptive statistical analysis was performed using SPSS (version 26.0) for the training and validation groups of each center. The chi‐square test was performed for qualitative variables and *t*‐test or rank‐sum test for continuous variables, with *p* < 0.05 indicating a significant difference. The predictive performance of the model was assessed using the area under the receiver operating characteristic (ROC) curve (AUC).

## 3 RESULTS

### 3.1 Patient characteristics

The basic characteristics of the patients in the three centers are listed in Table 2. The patients of each center were divided into an MIA group and an IAC group according to the pathological findings. For each center, there were no significant intergroup differences in the location of the pulmonary GGNs. There were no significant intergroup differences in smoking history at the second center, and gender and smoking history at the third center (*p* > 0.05). Conversely, there were significant intergroup differences in age and tumor diameter for each center. The results showed that nodal diameter is an important indicator for the clinical identification of IAC and MIA, and the analysis of the three‐center data revealed that the optimal nodal diameter cutoff value for differentiating the two subtypes is 10.5 mm.

**TABLE 2:** Baseline characteristics of the patients.

Clinical data	Cohort 1			Cohort 2			Cohort 3		
	IAC	MIA	*p*	IAC	MIA	P	IAC	MIA	*p*
Number of cases (cases)	305	234		123	166		101	70	
Age	63.71 ± 9.78	54.4 ± 12.4	**0.01**	62.4 ± 9.56	52.73 ± 11.69	**0.01**	57.78 ± 11.61	51.70 ± 13.10	**0.01**
Gender									
Male	110	54	**0.01**	44	42	**0.03**	35	19	0.30
Female	195	180		79	124		66	51	
Smoking history (cases)	68	24	**0.01**	27	21	0.54	5	3	0.84
Tumor length(cm)	1.60 ± 0.64	0.84 ± 0.23	**0.01**	1.52 ± 0.62	0.75 ± 0.24	**0.01**	1.29 ± 0.54	0.76 ± 0.24	**0.01**
location (left/right)	122/ 183	90/ 144	0.72	50/73	63/ 103	0.64	35/66	27/43	0.60

*Note*: The *t*‐test and rank‐sum test were performed. Continuous variables are expressed as means ± standard deviations. Categorical variables data include the number of patients. Results were compared by using the chi‐squared test. *P* < 0.05 indicates statistically significant difference (in bold).

Abbreviations: IAC, invasive adenocarcinoma; MIA, minimally invasive adenocarcinoma.

### 3.2 Predictive performance of the DL model

**FIGURE 4:**
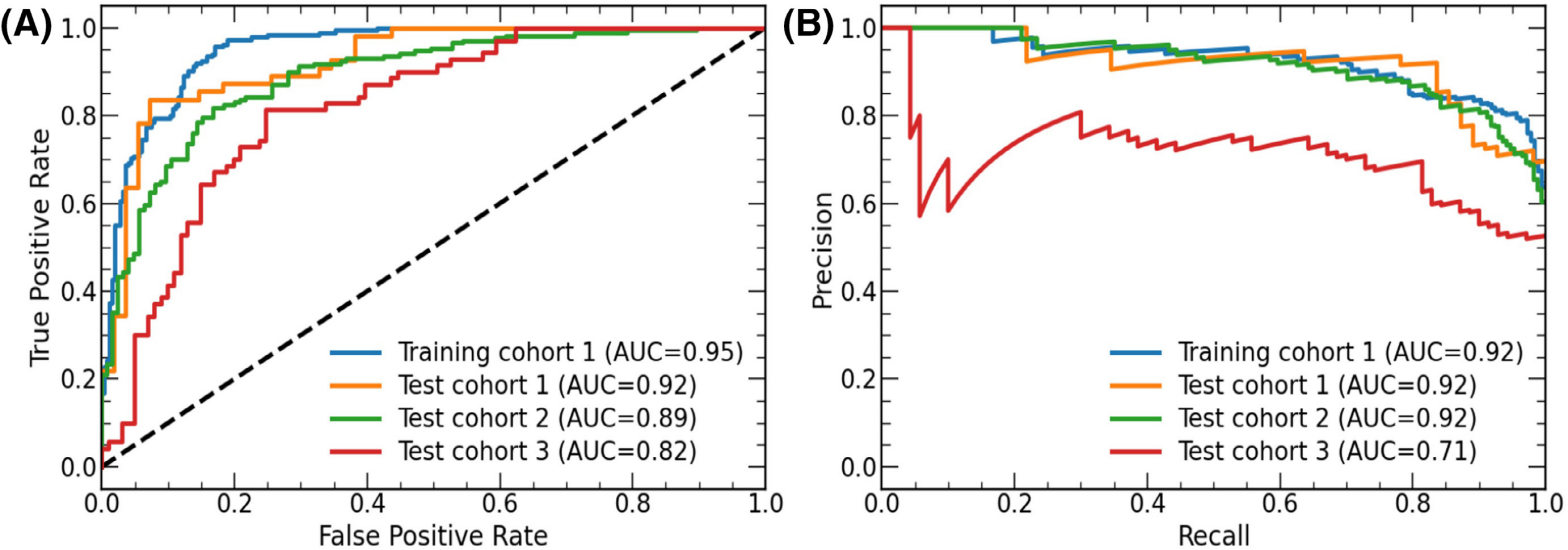
Receiver operating characteristics (ROC) and precision–recall (PR) curves for training, internal validation, and external validation.

**FIGURE 5:**
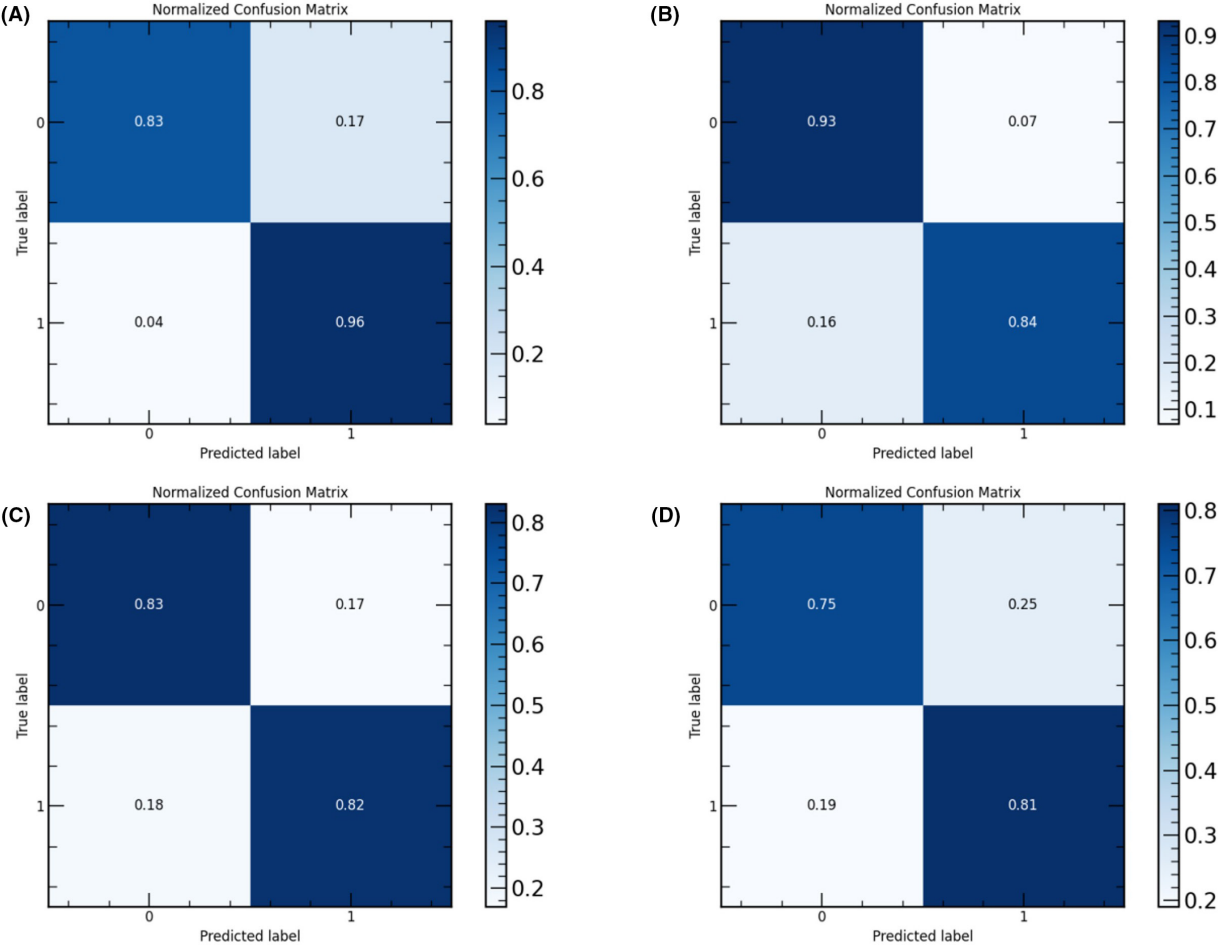
Corresponding confusion matrix for training, internal validation, and external validation.

For the training group, the radiomics‐based DL model achieved an accuracy of 89%, sensitivity of 98%, specificity of 81%, and AUC of 97%; for the internal validation group, it achieved an accuracy of 80%, sensitivity of 82%, specificity of 78%, and AUC of 92%. The ROC and precision–recall (PR) curves are shown in Figure 4, and the corresponding confusion matrix is presented in Figure 5. The ROC curve indicates that the AUC was lower for the external validation sets than for the internal validation and training sets. Moreover, the model performance differed for different external validation sets, that is, it was better for external validation set 2 than for external validation set 3 (*p* < 0.05). The PR curves show that the model had a similar performance for the training set, internal validation set, and external validation set 2, but a significantly lower performance for external validation set 3 (*p* < 0.05).

The main metrics of the model for the training, test, and external validation sets and their corresponding 95% confidence intervals were consistent with the ROC and PR curve analysis, that is, the model performance decreased in the order of internal validation set, external validation set 2, and external validation set 3. See Table 3 for more details of the performance metrics.

**TABLE 3:** Main metrics of the model for the training, test, and external validation sets and their corresponding 95% confidence intervals (CI 95%).

Datasets	Accuracy	Sensitivity	Specificity	F1	AUC
Training cohort 1	0.89 (0.86–0.92)	0.95 (0.90–0.99)	0.84 (0.78–0.90)	0.89 (0.86–0.92)	0.95 (0.93–0.97)
Internal validation cohort 1	0.88 (0.83–0.94)	0.84 (0.74–0.94)	0.93 (0.82–0.98)	0.88 (0.82–0.94)	0.92 (0.87–0.97)
External validation cohort 2	0.83 (0.79–0.87)	0.83 (0.74–0.94)	0.83 (0.70–0.91)	0.83 (0.78–0.87)	0.89 (0.85–0.92)
External validation cohort 3	0.78 (0.71–0.84)	0.80 (0.67–0.92)	0.77 (0.65–0.88)	0.78 (0.71–0.84)	0.82 (0.76–0.88)

## 4 DISCUSSION

This study demonstrated for the first time the application of a 3D medical image pretrained DL model combined with transfer learning to evaluate the pathological subtype of GGN‐type lung adenocarcinoma. Furthermore, the model exhibited excellent performance for internal and external validation sets, indicating the effectiveness and robustness of the proposed approach.

Most early lung adenocarcinomas appear as GGNs on thin‐slice CT images, and with the widespread availability of HRCT and low‐dose screening, the detection rate of pulmonary GGNs is increasing.^6^, ^17^ However, pulmonary GGNs are not unique to lung cancer because bacterial and fungal infections can also manifest as ground‐glass opacities. The Fleischner Society Guidelines for Management of Pulmonary Ground‐glass Nodules provide detailed treatment strategies.^18^ However, as regards observing and analyzing GGN edges, pleural depression, pleural retraction, lobulation, vacuoles, air bronchogram, vascular convergence signs, and other important details that can be used to help determine the nature of a pulmonary nodule, each physician's subjective interpretation of the signs and diagnostic experience may lead to very different diagnoses.

Multidisciplinary diagnosis and treatment (MDT) is a widely recognized option for providing many benefits to oncology patients through the multidimensional discussion and analysis of the disease, and it has been reported as capable of significantly improving the prognosis of patients.^19^ In the MDT mode, radiologists also rely on traditional imaging features for assessing pulmonary GGNs, but it has the advantage that the team members have rich diagnostic experience and thus achieve a high consistency in understanding the benign and malignant signs of pulmonary GGNs as well as a high accuracy in distinguishing between benign and malignant nodules. However, it is still difficult to distinguish between MIA and IAC because the two subtypes have significantly overlapped features in traditional imaging, although they differ significantly in surgical approach and prognosis.

The size of nodules is an important parameter for assessing the invasiveness of GGNs, and the optimal cutoff diameter is beneficial to the assessment of the invasiveness of lung adenocarcinoma.^6^, ^20^ Wang et al. reported an AUC for differentiating noninvasive nodes from IAC of 70.1% when using 10 mm as the cutoff diameter; after excluding AAH, the best cutoff diameter for differentiating noninvasive adenocarcinoma from IAC was 20 mm, and the corresponding AUC was 72%.^6^ Lee et al. reported that 14 mm was the best cutoff diameter for differentiating preinvasive MIA from IAC, with a sensitivity of 67% and specificity of 74%.^21^ In this study, there was a significant difference in the maximum tumor diameter between the MIA and IAC groups at each center. Joint data analysis of the three centers revealed that 10.5 mm was the best cutoff diameter for distinguishing between MIA and IAC, with a sensitivity of 76% and specificity of 88%, whereas the specificity reached more than 99% when the cutoff diameter was set to 15.5 mm. When the cutoff diameter was greater than 20 mm, the specificity reached 100%, but the sensitivity dropped to 16%. There is no consensus among studies on the use of nodal diameter to classify the degree of invasiveness.^6^ This discrepancy may be attributed to three main facts: (1) the current definitions of tumor diameter are inconsistent, with some using the longest tumor diameter as the tumor diameter and others using the sum of the longest tumor diameter plus half of the shortest diameter; (2) different researchers have varying degrees of error in the measurement of the diameter; and (3) the control group for IAC differs among different studies.

With the advent of the era of big data, the use of radiomics software to extract quantitative tumor features, and the use of artificial intelligence techniques to mine characteristic variables and construct quantitative prediction models provide new directions for developing analysis and decision‐making tools in imaging diagnosis. Chae et al. used artificial neural networks (ANNs) to build a radiomics‐based prediction model to identify preinvasive lesions (AAH/AIS) versus invasive lesions (MIA/IAC) in mGGN‐type lung adenocarcinoma and achieved excellent results (AUC = 98.1%).^22^ Wang et al. used a DL model combined with multitask learning to predict the invasiveness of GGN‐type lung adenocarcinoma, and the model performed well in distinguishing between MIA and IAC (AUC: 88.9%).^6^ Although ANNs solve the binary classification problem well, their generalizability for specific models is limited owing to overfitting and complex structures. Unlike previous studies that used natural image pretrained models to extract deep features, this study used a 3D medical image pretrained model. There are significant differences in dimensionality, structure, and intensity between natural images (2D) and medical images (3D). Therefore, 3D medical images cannot be effectively processed using natural image pretrained DL models, and the extracted features are not well adapted to medical images. The pretrained model used in this study was the first 3D medical image pretrained model with strong medical image‐feature extraction capability and robustness, which makes it convenient to transfer the model to the present task. The 3D deep transfer learning model constructed in this study displayed excellent performance in discriminating between MIA and IAC. Meanwhile, the model was externally validated using two external validation sets to avoid overfitting and the influence of complex structures. It displayed excellent performance on both internal and external data, indicating that it has good generalizability.

Nevertheless, although the model displayed high predictive performance on both internal and external data, there were significant differences in the ROC and PR curves between external validation sets 2 and 3 (*p* < 0.05), which may be attributed to the significant difference between external validation set 2 compared to other validation sets in the diameter of the IAC tumor (cohort 3: 1.29 cm vs. cohort 2: 1.52 cm vs. cohort 1: 1.60 cm). In other words, the significantly shorter diameter of the IAC tumor at the third center led to reduced model performance on validation set 3.

The significant difference in tumor diameter between the two external centers may be attributed to the following two main reasons. (1) The progression of AAH into AIS, MIA, and IAC is a dynamic continuous process involving multiple genes, in which AAH and AIS can gradually develop into MIA and IAC.^5^ Patients often have no obvious clinical symptoms, and these diseases are mainly detected by physical examinations. The coverage of universal health examinations in first‐tier provincial capitals (such as the city where the cohort hospital with external validation set 3 is located) is higher than that in county hospitals (such as the cities where the hospitals with the internal validation set and external validation set 2 are located). Consequently, it is easier to detect patients with lung cancer in the early stage in first‐tier provincial capitals. (2) County patients have a long physical examination cycle, and the time interval between first‐nodule detection and surgical resection may be even longer. Consequently, the IAC tumor—which is more invasive than other‐stage tumors—may be larger in diameter for county patients. In addition, the ROC and PR curves of the model were different between the training, internal validation set, and external validation sets, possibly because the PR curves were more sensitive to the imbalance of positive and negative samples and focused more on positive samples, whereas the ratio of positive to negative samples was not the same for each validation set (cohort 1‐train: 0.73 vs. cohort 1‐test: 1.00 vs. cohort 2: 1.37 vs. cohort 3: 0.70), thereby resulting in a certain degree of inconsistency between the PR and ROC curves.

This study had several limitations. First, it was a retrospective study with inherent bias, and thus prospective applications are needed to ascertain the robustness of the present results further. Second, ROIs were all delineated manually, which was time‐consuming and labor‐intensive. Third, although there were two cohort hospitals as external centers to provide data, the data from the three centers were still focused on one racial group, thus the predictive performance of the model in other racial groups remains to be validated.

In future research, we will include a larger sample of patients and develop an artificial intelligence‐based tool for the automatic delineation of ROIs so that the model will improve to become more suitable for large samples. Further, a clinical risk model for lung cancer will be developed to facilitate the development of personalized clinical treatment plans.

This study demonstrated that by jointly using a model pretrained on 3D medical images and transfer learning, it is possible to discriminate between IAC and MIA effectively, although both manifest as pulmonary GGNs. The results verify that the approach can provide a noninvasive, low‐cost, and reproducible method for preoperative prediction in a clinical setting.

## AUTHOR CONTRIBUTIONS

**chunlong fu:** Conceptualization (lead); data curation (lead); formal analysis (lead); investigation (lead); methodology (equal); software (equal); validation (lead); writing – original draft (lead). **zebin yang:** Data curation (lead); formal analysis (equal); investigation (equal); methodology (equal); resources (equal); validation (lead); writing – original draft (equal). **ping li:** Data curation (equal); formal analysis (equal); methodology (equal); software (equal); validation (equal); writing – original draft (equal). **kangfei shan:** Data curation (equal); formal analysis (equal); investigation (equal); resources (equal); software (equal); validation (equal). **meikang wu:** Data curation (equal); formal analysis (equal); investigation (equal); software (equal); validation (equal). **jieping xu:** Data curation (equal); investigation (equal); methodology (equal); software (equal); validation (equal); visualization (equal). **chijun ma:** Data curation (equal); formal analysis (equal); investigation (equal); software (equal); visualization (equal). **fanghong luo:** Data curation (equal); formal analysis (equal); investigation (equal); validation (equal); visualization (equal). **long zhou:** Conceptualization (lead); formal analysis (equal); investigation (equal); methodology (lead); project administration (equal); software (equal); supervision (equal); validation (equal); visualization (equal); writing – original draft (lead); writing – review and editing (lead). **Ji‐Hong Sun:** Conceptualization (equal); funding acquisition (equal); project administration (equal); resources (lead); supervision (lead); writing – review and editing (lead). **fenhua zhao:** Conceptualization (equal); funding acquisition (lead); project administration (lead); resources (lead); supervision (lead); writing – review and editing (lead).

## FUNDING INFORMATION

This study was supported by Jinhua Science and Technology Bureau (2022–3‐015).

## CONFLICT OF INTEREST STATEMENT

The authors declare that they have no known competing financial interests or personal relationships that could have appeared to influence the work reported in this paper.

## ETHICS STATEMENT

The study was approved by institutional ethics board of Sir Run Run Shaw Hospital, Zhejiang University School of Medicine (approval number: 0260) and Affiliated Dongyang Hospital of Wenzhou Medical University, (approval number: 2023‐YX‐038) (Yiwu Tianxiang Medical Eastern Hospital and Affiliated Dongyang Hospital of Wenzhou Medical University share an ethics committee) and individual consent for this retrospective analysis was waived.

## Data Availability

The data that support the findings of this study are available from the corresponding author, F.Z, upon reasonable request.
